# 3-Ethyl­sulfinyl-5-iodo-2-phenyl-1-benzofuran

**DOI:** 10.1107/S1600536810024190

**Published:** 2010-06-26

**Authors:** Pil Ja Seo, Hong Dae Choi, Byeng Wha Son, Uk Lee

**Affiliations:** a Department of Chemistry, Dongeui University, San 24 Kaya-dong Busanjin-gu, Busan 614-714, Republic of Korea; b Department of Chemistry, Pukyong National University, 599-1 Daeyeon 3-dong, Nam-gu, Busan 608-737, Republic of Korea

## Abstract

In the title compound, C_16_H_13_IO_2_S, the phenyl ring is rotated out of the benzofuran plane, as indicated by the dihedral angle of 32.56 (6)°. The crystal structure is stabilized by an I⋯O halogen inter­action [3.200 (2) Å].

## Related literature

For the crystal structures of similar 2-aryl-3-ethyl­sulfinyl-5-halo-1-benzofuran derivatives, see: Choi *et al.* (2010*a* ▶,*b* ▶). For the pharmacological activity of benzofuran compounds, see: Aslam *et al.* (2006 ▶); Galal *et al.* (2009 ▶); Khan *et al.* (2005 ▶). For natural products with benzofuran rings, see: Akgul & Anil (2003 ▶); Soekamto *et al.* (2003 ▶). For a review of halogen bonding, see: Politzer *et al.* (2007 ▶).
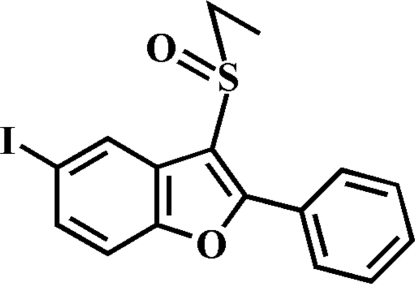


## Experimental

### 

#### Crystal data

C_16_H_13_IO_2_S
*M* *_r_* = 396.22
Orthorhombic, 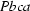

*a* = 11.7297 (4) Å
*b* = 7.4560 (2) Å
*c* = 34.0313 (9) Å
*V* = 2976.26 (15) Å^3^
*Z* = 8
Mo *K*α radiation
μ = 2.29 mm^−1^
*T* = 296 K
0.02 × 0.02 × 0.02 mm

#### Data collection

Bruker SMART APEXII CCD diffractometer
Absorption correction: multi-scan (*SADABS*; Bruker, 2009 ▶) *T*_min_ = 0.954, *T*_max_ = 0.967
14370 measured reflections
3415 independent reflections
3106 reflections with *I* > 2σ(*I*)
*R*_int_ = 0.027

#### Refinement

*R*[*F* ^2^ > 2σ(*F* ^2^)] = 0.028
*wR*(*F* ^2^) = 0.068
*S* = 1.15
3415 reflections
182 parameters
H-atom parameters constrained
Δρ_max_ = 0.55 e Å^−3^
Δρ_min_ = −0.97 e Å^−3^

### 

Data collection: *APEX2* (Bruker, 2009 ▶); cell refinement: *SAINT* (Bruker, 2009 ▶); data reduction: *SAINT*; program(s) used to solve structure: *SHELXS97* (Sheldrick, 2008 ▶); program(s) used to refine structure: *SHELXL97* (Sheldrick, 2008 ▶); molecular graphics: *ORTEP-3* (Farrugia, 1997 ▶) and *DIAMOND* (Brandenburg, 1998 ▶); software used to prepare material for publication: *SHELXL97*.

## Supplementary Material

Crystal structure: contains datablocks global, I. DOI: 10.1107/S1600536810024190/kp2264sup1.cif

Structure factors: contains datablocks I. DOI: 10.1107/S1600536810024190/kp2264Isup2.hkl

Additional supplementary materials:  crystallographic information; 3D view; checkCIF report

## Figures and Tables

**Fig. 1.**
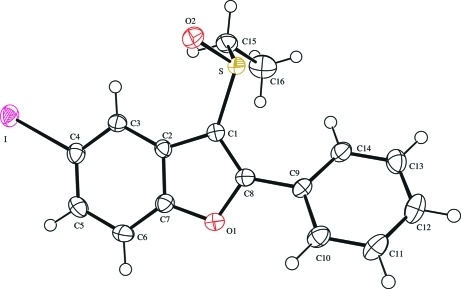
The molecular structure of the title compound with the atom numbering scheme. Displacement ellipsoids are drawn at the 50% probability level. H atoms are presented as a small spheres of arbitrary radius.

**Fig. 2.**
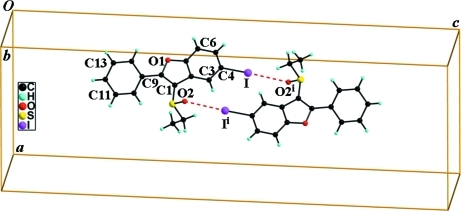
I···O interactions (dotted lines) in the crystal structure of the title compound. [Symmetry codes: (i) - x + 1, - y, - z + 1 .]

## supplementary crystallographic information

### Comment

The compounds involving benzofuran skeleton show various pharmacological properties such as antifungal (Aslam *et al.*, 2006), antitumor and antiviral (Galal *et al.*, 2009), antimicrobial (Khan *et al.*, 2005) activities.

These compounds widely occur in nature (Akgul & Anil, 2003; Soekamto *et al.*, 2003). As a part of our ongoing  studies of the effect of side chain substituents on the solid state structures of 2-aryl-3-ethylsulfinyl-5-halo-1-benzofuran analogues (Choi*et al.*, 2010*a*,*b*), we report the crystal structure of the title compound (Fig. 1).

The benzofuran unit is essentially planar, with a mean deviation of 0.005 (2) Å from the least-squares plane defined by the nine constituent atoms. The dihedral angle formed by the benzofuran plane and the phenyl ring is 32.56 (6)°. The crystal packing (Fig. 2) is stabilized by I···O halogen–bonding interactions between the iodine and the oxygen of the S═O unit [I···O2^i^ = 3.200 (2) Å; C4–I···O2^i^ = 176.44 (8)°] (Politzer *et al.*, 2007).

### Experimental

77% 3-chloroperoxybenzoic acid (247 mg, 1.1 mmol) was added in small portions to a stirred solution of 3-ethylsulfanyl-5-iodo-2-phenyl-1-benzofuran (380 mg, 1.0 mmol) in dichloromethane (30 mL) at 273 K. After being stirred at room temperature for 4 h, the mixture was washed with saturated sodium bicarbonate solution and the organic layer was separated, dried over magnesium sulfate, filtered and concentrated at reduced pressure. The residue was purified by column chromatography (hexane-ethyl acetate, 1:1 v/v) to afford the title compound as a colourless solid [yield 79%, m.p. 419-420 K; *R*_f_ = 0.5 (hexane-ethyl acetate, 1:1 v/v)]. Single crystals suitable for X-ray diffraction were prepared by slow evaporation of a solution of the title compound in acetone at room temperature.

### Refinement

All H atoms were positioned geometrically and refined using a riding model, with C–H = 0.95 Å for aryl,  0.97 Å for methylene  and 0.96 Å for methyl H atoms.  *U*_iso_(H) = 1.2*U*_eq_(C) for aryl and methylene H atoms, and 1.5*U*_eq_(C) for methyl H atoms.

### Crystal data

**Table:**

C_16_H_13_IO_2_S	*F*(000) = 1552
*M**_r_* = 396.22	*D*_x_ = 1.769 Mg m^−^^3^
Orthorhombic, *P**b**c**a*	Mo *K*α radiation, λ = 0.71073 Å
Hall symbol: -P 2ac 2ab	Cell parameters from 8139 reflections
*a* = 11.7297 (4) Å	θ = 2.4–27.5°
*b* = 7.4560 (2) Å	µ = 2.29 mm^−^^1^
*c* = 34.0313 (9) Å	*T* = 296 K
*V* = 2976.26 (15) Å^3^	Block, colourless
*Z* = 8	0.02 × 0.02 × 0.02 mm

### Data collection

**Table:**

Bruker SMART APEXII CCD diffractometer	3415 independent reflections
Radiation source: rotating anode	3106 reflections with *I* > 2σ(*I*)
graphite multilayer	*R*_int_ = 0.027
Detector resolution: 10.0 pixels mm^-1^	θ_max_ = 27.5°, θ_min_ = 2.1°
φ and ω scans	*h* = −15→9
Absorption correction: multi-scan (*SADABS*; Bruker, 2009)	*k* = −8→9
*T*_min_ = 0.954, *T*_max_ = 0.967	*l* = −43→44
14370 measured reflections	

### Refinement

**Table:**

Refinement on *F*^2^	Primary atom site location: structure-invariant direct methods
Least-squares matrix: full	Secondary atom site location: difference Fourier map
*R*[*F*^2^ > 2σ(*F*^2^)] = 0.028	Hydrogen site location: difference Fourier map
*wR*(*F*^2^) = 0.068	H-atom parameters constrained
*S* = 1.15	*w* = 1/[σ^2^(*F*_o_^2^) + (0.026*P*)^2^ + 3.2745*P*] where *P* = (*F*_o_^2^ + 2*F*_c_^2^)/3
3415 reflections	(Δ/σ)_max_ = 0.002
182 parameters	Δρ_max_ = 0.55 e Å^−^^3^
0 restraints	Δρ_min_ = −0.97 e Å^−^^3^

### Special details

**Table:**

Geometry. All esds (except the esd in the dihedral angle between two l.s. planes) are estimated using the full covariance matrix. The cell esds are taken into account individually in the estimation of esds in distances, angles and torsion angles; correlations between esds in cell parameters are only used when they are defined by crystal symmetry. An approximate (isotropic) treatment of cell esds is used for estimating esds involving l.s. planes.
Refinement. Refinement of F^2^ against ALL reflections. The weighted R-factor wR and goodness of fit S are based on F^2^, conventional R-factors R are based on F, with F set to zero for negative F^2^. The threshold expression of F^2^ > 2sigma(F^2^) is used only for calculating R-factors(gt) etc. and is not relevant to the choice of reflections for refinement. R-factors based on F^2^ are statistically about twice as large as those based on F, and R- factors based on ALL data will be even larger.

### Fractional atomic coordinates and isotropic or equivalent isotropic displacement parameters (Å^2^)

**Table:**

	*x*	*y*	*z*	*U*_iso_*/*U*_eq_	
I	0.337055 (15)	0.11075 (3)	0.528788 (5)	0.03128 (7)	
S	0.56486 (5)	0.15154 (8)	0.360798 (17)	0.02225 (13)	
O1	0.23461 (14)	0.2567 (2)	0.35329 (5)	0.0259 (4)	
O2	0.58668 (16)	−0.0183 (3)	0.38324 (6)	0.0318 (4)	
C1	0.4196 (2)	0.2055 (3)	0.36723 (7)	0.0217 (5)	
C2	0.3566 (2)	0.1963 (3)	0.40373 (7)	0.0212 (5)	
C3	0.3845 (2)	0.1631 (3)	0.44285 (7)	0.0239 (5)	
H3	0.4594	0.1420	0.4506	0.029*	
C4	0.2959 (2)	0.1628 (4)	0.46975 (7)	0.0261 (5)	
C5	0.1832 (2)	0.1945 (4)	0.45888 (8)	0.0296 (6)	
H5	0.1263	0.1937	0.4779	0.036*	
C6	0.1550 (2)	0.2274 (4)	0.41965 (8)	0.0288 (6)	
H6	0.0802	0.2483	0.4118	0.035*	
C7	0.2435 (2)	0.2273 (3)	0.39332 (7)	0.0244 (5)	
C8	0.34345 (19)	0.2429 (3)	0.33829 (7)	0.0219 (5)	
C9	0.3539 (2)	0.2680 (3)	0.29579 (7)	0.0226 (5)	
C10	0.2650 (2)	0.2139 (3)	0.27091 (8)	0.0289 (5)	
H10	0.1990	0.1639	0.2814	0.035*	
C11	0.2760 (3)	0.2353 (4)	0.23078 (8)	0.0353 (6)	
H11	0.2171	0.1990	0.2143	0.042*	
C12	0.3734 (3)	0.3100 (4)	0.21470 (8)	0.0346 (6)	
H12	0.3802	0.3226	0.1876	0.042*	
C13	0.4609 (3)	0.3660 (4)	0.23919 (8)	0.0320 (6)	
H13	0.5261	0.4176	0.2286	0.038*	
C14	0.4512 (2)	0.3451 (3)	0.27951 (7)	0.0273 (5)	
H14	0.5101	0.3829	0.2958	0.033*	
C15	0.6250 (2)	0.3317 (4)	0.38974 (8)	0.0314 (6)	
H15A	0.5900	0.3315	0.4156	0.038*	
H15B	0.7060	0.3106	0.3931	0.038*	
C16	0.6077 (3)	0.5131 (4)	0.37097 (10)	0.0434 (7)	
H16A	0.6474	0.5172	0.3463	0.065*	
H16B	0.6369	0.6047	0.3880	0.065*	
H16C	0.5278	0.5327	0.3666	0.065*	

### Atomic displacement parameters (Å^2^)

**Table:**

	*U*^11^	*U*^22^	*U*^33^	*U*^12^	*U*^13^	*U*^23^
I	0.03590 (12)	0.03674 (12)	0.02121 (10)	0.00703 (7)	0.00679 (6)	0.00190 (7)
S	0.0199 (3)	0.0276 (3)	0.0193 (3)	0.0033 (2)	0.0005 (2)	0.0016 (2)
O1	0.0206 (8)	0.0319 (9)	0.0251 (8)	0.0007 (7)	−0.0023 (7)	0.0016 (7)
O2	0.0311 (10)	0.0325 (10)	0.0318 (10)	0.0093 (8)	0.0016 (8)	0.0083 (8)
C1	0.0209 (11)	0.0235 (12)	0.0208 (11)	−0.0001 (9)	0.0002 (9)	0.0007 (9)
C2	0.0212 (11)	0.0198 (12)	0.0226 (11)	0.0016 (9)	0.0029 (9)	0.0022 (9)
C3	0.0223 (12)	0.0251 (12)	0.0242 (12)	0.0042 (10)	0.0013 (9)	0.0007 (10)
C4	0.0301 (13)	0.0252 (12)	0.0231 (12)	0.0039 (10)	0.0038 (10)	0.0010 (10)
C5	0.0262 (13)	0.0328 (15)	0.0299 (13)	0.0005 (11)	0.0081 (10)	−0.0004 (11)
C6	0.0192 (12)	0.0349 (14)	0.0322 (14)	0.0021 (10)	−0.0003 (10)	0.0002 (11)
C7	0.0242 (11)	0.0233 (12)	0.0256 (12)	0.0008 (10)	−0.0012 (10)	0.0024 (10)
C8	0.0199 (11)	0.0208 (11)	0.0250 (12)	−0.0001 (9)	−0.0018 (9)	0.0003 (9)
C9	0.0256 (12)	0.0204 (12)	0.0217 (12)	0.0026 (9)	−0.0043 (9)	0.0008 (9)
C10	0.0313 (14)	0.0251 (13)	0.0304 (13)	−0.0039 (11)	−0.0077 (11)	0.0032 (10)
C11	0.0465 (17)	0.0304 (14)	0.0289 (13)	−0.0046 (12)	−0.0144 (12)	0.0017 (11)
C12	0.0563 (18)	0.0251 (13)	0.0226 (12)	0.0014 (13)	−0.0035 (12)	0.0027 (10)
C13	0.0372 (15)	0.0298 (14)	0.0291 (13)	0.0016 (11)	0.0048 (11)	0.0067 (11)
C14	0.0282 (13)	0.0269 (13)	0.0269 (13)	−0.0005 (10)	−0.0055 (10)	0.0043 (10)
C15	0.0236 (12)	0.0421 (16)	0.0284 (13)	−0.0054 (12)	−0.0048 (10)	−0.0047 (12)
C16	0.0392 (16)	0.0340 (16)	0.057 (2)	−0.0074 (13)	−0.0056 (15)	−0.0078 (14)

### Geometric parameters (Å, °)

**Table:**

I—C4	2.102 (2)	C8—C9	1.464 (3)
I—O2^i^	3.200 (2)	C9—C14	1.393 (4)
S—O2	1.500 (2)	C9—C10	1.402 (3)
S—C1	1.764 (2)	C10—C11	1.381 (4)
S—C15	1.809 (3)	C10—H10	0.9300
O1—C8	1.379 (3)	C11—C12	1.384 (4)
O1—C7	1.384 (3)	C11—H11	0.9300
C1—C8	1.359 (3)	C12—C13	1.386 (4)
C1—C2	1.447 (3)	C12—H12	0.9300
C2—C7	1.392 (3)	C13—C14	1.385 (4)
C2—C3	1.393 (3)	C13—H13	0.9300
C3—C4	1.385 (3)	C14—H14	0.9300
C3—H3	0.9300	C15—C16	1.510 (4)
C4—C5	1.393 (4)	C15—H15A	0.9700
C5—C6	1.397 (4)	C15—H15B	0.9700
C5—H5	0.9300	C16—H16A	0.9600
C6—C7	1.372 (4)	C16—H16B	0.9600
C6—H6	0.9300	C16—H16C	0.9600
			
C4—I—O2^i^	176.44 (8)	C14—C9—C8	121.0 (2)
O2—S—C1	107.1 (1)	C10—C9—C8	119.8 (2)
O2—S—C15	106.4 (1)	C11—C10—C9	119.6 (3)
C1—S—C15	98.0 (1)	C11—C10—H10	120.2
C8—O1—C7	106.4 (2)	C9—C10—H10	120.2
C8—C1—C2	107.2 (2)	C10—C11—C12	121.0 (3)
C8—C1—S	126.3 (2)	C10—C11—H11	119.5
C2—C1—S	126.1 (2)	C12—C11—H11	119.5
C7—C2—C3	119.8 (2)	C11—C12—C13	119.6 (3)
C7—C2—C1	105.1 (2)	C11—C12—H12	120.2
C3—C2—C1	135.1 (2)	C13—C12—H12	120.2
C4—C3—C2	117.1 (2)	C14—C13—C12	120.1 (3)
C4—C3—H3	121.4	C14—C13—H13	120.0
C2—C3—H3	121.4	C12—C13—H13	120.0
C3—C4—C5	122.5 (2)	C13—C14—C9	120.5 (2)
C3—C4—I	117.4 (2)	C13—C14—H14	119.8
C5—C4—I	120.2 (2)	C9—C14—H14	119.8
C4—C5—C6	120.6 (2)	C16—C15—S	112.5 (2)
C4—C5—H5	119.7	C16—C15—H15A	109.1
C6—C5—H5	119.7	S—C15—H15A	109.1
C7—C6—C5	116.4 (2)	C16—C15—H15B	109.1
C7—C6—H6	121.8	S—C15—H15B	109.1
C5—C6—H6	121.8	H15A—C15—H15B	107.8
C6—C7—O1	125.8 (2)	C15—C16—H16A	109.5
C6—C7—C2	123.7 (2)	C15—C16—H16B	109.5
O1—C7—C2	110.4 (2)	H16A—C16—H16B	109.5
C1—C8—O1	110.8 (2)	C15—C16—H16C	109.5
C1—C8—C9	133.4 (2)	H16A—C16—H16C	109.5
O1—C8—C9	115.7 (2)	H16B—C16—H16C	109.5
C14—C9—C10	119.2 (2)		
			
O2—S—C1—C8	−128.5 (2)	C1—C2—C7—O1	−0.8 (3)
C15—S—C1—C8	121.5 (2)	C2—C1—C8—O1	−0.7 (3)
O2—S—C1—C2	43.2 (2)	S—C1—C8—O1	172.33 (17)
C15—S—C1—C2	−66.8 (2)	C2—C1—C8—C9	−179.8 (3)
C8—C1—C2—C7	0.9 (3)	S—C1—C8—C9	−6.8 (4)
S—C1—C2—C7	−172.14 (19)	C7—O1—C8—C1	0.2 (3)
C8—C1—C2—C3	179.7 (3)	C7—O1—C8—C9	179.5 (2)
S—C1—C2—C3	6.7 (4)	C1—C8—C9—C14	−33.4 (4)
C7—C2—C3—C4	0.0 (4)	O1—C8—C9—C14	147.5 (2)
C1—C2—C3—C4	−178.7 (3)	C1—C8—C9—C10	146.6 (3)
C2—C3—C4—C5	−0.2 (4)	O1—C8—C9—C10	−32.5 (3)
C2—C3—C4—I	179.54 (19)	C14—C9—C10—C11	1.0 (4)
C3—C4—C5—C6	0.4 (4)	C8—C9—C10—C11	−179.0 (2)
I—C4—C5—C6	−179.4 (2)	C9—C10—C11—C12	−0.2 (4)
C4—C5—C6—C7	−0.3 (4)	C10—C11—C12—C13	−0.7 (5)
C5—C6—C7—O1	180.0 (2)	C11—C12—C13—C14	0.8 (4)
C5—C6—C7—C2	0.1 (4)	C12—C13—C14—C9	0.0 (4)
C8—O1—C7—C6	−179.5 (3)	C10—C9—C14—C13	−0.9 (4)
C8—O1—C7—C2	0.4 (3)	C8—C9—C14—C13	179.1 (2)
C3—C2—C7—C6	0.0 (4)	O2—S—C15—C16	−179.1 (2)
C1—C2—C7—C6	179.1 (2)	C1—S—C15—C16	−68.5 (2)
C3—C2—C7—O1	−179.8 (2)		

Symmetry codes: (i) −*x*+1, −*y*, −*z*+1.
